# Feasibility and Acceptability of Craniosacral Therapy and Respiratory Training in Mothers of Children with Disabilities: Protocol for a Three-Arm Non-Randomized Controlled Pilot and Feasibility Study

**DOI:** 10.3390/healthcare14183093

**Published:** 2026-09-19

**Authors:** Aneta Gutowska, Jan Szewieczek, Marcin Sikora, Szymon Siatkowski, Marcin Żak, Diana Celebańska, Anna Zwierzchowska

**Affiliations:** 1 Institute of Sport Sciences, The Jerzy Kukuczka Academy of Physical Education in Katowice, 40-065 Katowice, Poland; 2 Department of Geriatrics, Faculty of Health Sciences in Katowice, Medical University of Silesia, 40-752 Katowice, Poland; jszewieczek@sum.edu.pl

**Keywords:** quality of life, stress, mother of a child with a disability, craniosacral therapy, respiratory training

## Abstract

Introduction: Mothers of children with disabilities may experience substantial and prolonged psychophysical burden, which can negatively affect psychological well-being, health-related quality of life, and physical functioning. This study describes the protocol of a pilot and feasibility study evaluating the feasibility and acceptability of two distinct non-pharmacological interventions—craniosacral therapy (CST) and respiratory training (RT)—in mothers of children with disabilities. Methods and Analysis: This completed, retrospectively registered, three-arm, parallel-group, non-randomized controlled pilot and feasibility study enrolled 32 mothers, allocated by enrolment timing and project logistics to craniosacral therapy (CST; *n* = 12), respiratory training (RT; *n* = 10), or no-intervention control (CG; *n* = 10). The interventions lasted 8 weeks. Feasibility outcomes included recruitment, consent, retention, attendance/adherence, outcome completeness, fidelity indicators, withdrawals, and reported adverse events; acceptability was assessed indirectly because no dedicated acceptability instrument was administered. Clinical outcomes were exploratory. Analyses estimated pre–post change and between-group differences, with confidence intervals reported where supported by the analyses. Recruitment occurred from May to December 2025, follow-up ended on 30 April 2026, and outcome data were analyzed before submission. Ethics approval preceded enrolment (2-XII/2024); registration was retrospective (ANZCTR ACTRN12626000148370; 5 February 2026). Discussion: This pilot and feasibility study provides a structured framework for evaluating CST and RT in mothers of children with disabilities. The study is intended to inform methodological procedures, outcome selection, and the design of a future adequately powered randomized controlled trial rather than establish the clinical effectiveness or safety of either intervention.

## 1. Introduction

### 1.1. Background and Rationale

According to data from Statistics Poland (GUS, 2022/2023), approximately 4.3% of children in Poland have an official disability certificate. A child’s disability constitutes a substantial burden on the entire family system, affecting its functioning in psychological, physical, and social dimensions. In the literature, this experience is described as “intensive mothering” or “hyper mothering,” referring to an increased and prolonged caregiving burden associated with elevated levels of stress and emotional strain [1]. Psychological and physiological stress plays a significant role in determining health status, and its importance is particularly evident among parents of children with severe disabilities who provide lifelong care. Parenting a child with a disability is often described as a state of chronic stress that requires continuous adaptation to non-normative life events, which may negatively affect caregivers’ physical and mental health [2]. Evidence from the literature indicates that this population experiences reduced quality of life and accelerated aging processes, both cognitively and somatically, compared with parents of healthy children, which have been associated with prolonged exposure to stressors [1,3,4].

Therefore, there is a justified need for research focusing on the well-being of caregivers of children with disabilities, aimed at identifying psychological and functional factors that may protect this population against deterioration of cognitive and somatic functioning. Despite growing awareness of this issue, evidence regarding effective interventions designed to improve well-being and, consequently, quality of life in this population remains limited.

The primary purpose of this pilot and feasibility study was to assess the feasibility and acceptability of implementing two distinct non-pharmacological interventions- craniosacral therapy (CST) and respiratory training (RT)- in mothers of children with disabilities. A secondary purpose was to obtain preliminary estimates of changes in selected clinical outcomes to inform the design of a future adequately powered trial.

Craniosacral therapy (CST) is used in complementary practice for conditions including musculoskeletal pain and headache; however, evidence supporting its clinical effectiveness remains limited and inconsistent [5,6,7,8]. Within the conceptual framework of CST, gentle manual contact is proposed to influence perceived tension and relaxation [9], but these proposed mechanisms have not been conclusively established. Some trials have reported potential improvements in pain or selected patient-reported outcomes [6,7,10,11,12], whereas systematic reviews have emphasized heterogeneity, methodological limitations, and low certainty of evidence [10]. CST was therefore included in the present pilot as an exploratory intervention rather than as an established treatment.

Although craniosacral therapy (CST) is used as a complementary, non-pharmacological intervention, the evidence regarding its clinical effectiveness remains limited and inconsistent. Some previous randomized trials and systematic reviews have reported potential improvements in pain, disability, and selected patient-reported outcomes; however, methodological limitations and heterogeneity across studies restrict the certainty and generalizability of these findings. A recent systematic review and meta-analysis of randomized controlled trials found no statistically significant or clinically relevant benefits for several musculoskeletal and non-musculoskeletal conditions and highlighted the low or very low certainty of evidence for several outcomes [10]. Therefore, CST should not be regarded as an intervention with established clinical effectiveness. In the present pilot and feasibility study, CST was investigated as an exploratory non-pharmacological intervention, with the aim of assessing its feasibility and acceptability and obtaining preliminary estimates of potential changes in selected psychophysical outcomes rather than confirming therapeutic efficacy.

Similarly, scientific evidence indicates that controlled breathing practices may have potential benefits for stress management and well-being enhancement. Controlled breathing has been practiced for centuries in Eastern cultures, particularly in the form of pranayama within the yoga tradition [13]. Contemporary evidence suggests that even a daily five-minute practice of cyclic sighing may be a promising and effective strategy for coping with stress [14]. It has also been demonstrated that regular slow-breathing practice performed over 12 weeks significantly reduces psychological stress, regardless of the breathing ratio employed [15].

### 1.2. Conceptual Framework

The conceptual framework of the study is based on the assumption that the prolonged caregiving demands experienced by mothers of children with disabilities may be associated with multidimensional psychophysical burden, including reduced psychological well-being and health-related quality of life, musculoskeletal complaints, altered muscle and soft-tissue tension, and disturbances in respiratory and sleep-related functioning. Chronic caregiving demands may also be accompanied by physiological stress responses, which can be explored through biochemical markers such as salivary cortisol.

The two interventions investigated in this study were selected as distinct non-pharmacological approaches potentially addressing different components of this multidimensional burden. Craniosacral therapy (CST) was included as a gentle, therapist-delivered somatic intervention aimed at promoting relaxation and modulating perceived and physical tension. Respiratory training (RT), incorporating inspiratory muscle training, was included as an active, home-based intervention aimed primarily at improving respiratory function while potentially supporting relaxation and psychophysical well-being.

Within this conceptual framework, feasibility and acceptability constitute the primary focus of the pilot study. Clinical outcomes are considered exploratory and include psychological well-being assessed using the World Health Organization Five Well-Being Index (WHO-5), health-related quality of life assessed using the 36-Item Short Form Health Survey (SF-36), musculoskeletal complaints assessed using the Nordic Musculoskeletal Questionnaire for symptoms experienced during the previous 6 months (NMQ-6) and the previous 7 days (NMQ-7), pain intensity assessed using the Visual Analogue Scale (VAS), biomechanical measures of spinal mobility and the mechanical properties of paraspinal soft tissues, respiratory function, sleep-related parameters, and salivary cortisol concentration as a biochemical marker associated with the physiological stress response.

The assessment of these multidimensional outcomes is intended to explore potential patterns of change associated with CST and RT rather than to provide confirmatory evidence of intervention effectiveness. The resulting preliminary estimates may help identify potentially responsive outcome measures and inform the methodological design, outcome selection, and sample-size planning of a future adequately powered randomized controlled trial.

### 1.3. Study Objectives

The primary objective of this pilot and feasibility study was to assess the feasibility and acceptability of implementing two non-pharmacological interventions—craniosacral therapy (CST) and respiratory training (RT)—in mothers of children with disabilities. Feasibility and acceptability were considered in relation to recruitment and retention, intervention attendance and adherence, completeness of outcome assessments, intervention fidelity, withdrawals, and adverse events.

A secondary objective was to obtain preliminary estimates of potential changes in participants’ psychological well-being and health-related quality of life and to explore differences between the craniosacral therapy, respiratory training, and control groups. The registered primary clinical outcomes were spinal mobility assessed using the IDIAG M360 system and respiratory function assessed using maximal inspiratory pressure (MIP). Secondary clinical outcomes included salivary cortisol concentration, myotonometric measures of tissue tone and stiffness, musculoskeletal symptoms, health-related quality of life assessed using the 36-Item Short Form Health Survey (SF-36), and psychological well-being assessed using the World Health Organization Five Well-Being Index (WHO-5). Musculoskeletal complaints and pain, as well as biomechanical, respiratory, sleep-related, and biochemical measures, were considered secondary or exploratory outcomes.

Given the pilot nature of the study and its limited sample size, analyses of clinical outcomes were intended to provide preliminary estimates of intervention effects and their corresponding confidence intervals rather than confirmatory evidence of intervention effectiveness. These preliminary findings were intended to inform the design, selection of outcomes, and methodological procedures of a future adequately powered randomized controlled trial.

### 1.4. Pilot Study Design

This research was funded by the Minister of Science under the ‘Regional Initiative of Excellence’ programme, no. RID/SP/0053/2024/01 “Promoting Healthy Lifestyles and Extending Lifespan” (Contract No. RID/SP/0053/2024/01). The mission of the research teams implementing the mini grants is to contribute to the early detection of abnormalities in human functioning, particularly in the areas of motor control, compensation of motor impairments, and support of adaptive mechanisms influencing the physical and mental health of society.

This study was designed as a three-arm, parallel, non-randomized controlled pilot and feasibility study comprising two intervention groups and one control group. Participants were allocated to study groups non-randomly according to the sequence of enrolment and organizational requirements of the project, as described in detail in the Group Allocation section.

The primary focus of the pilot study was to assess the feasibility and acceptability of the study procedures and interventions. Feasibility and acceptability were evaluated pragmatically using recruitment, consent, retention, intervention attendance and adherence, completeness of outcome assessments, intervention fidelity, withdrawals, and adverse events. No formal qualitative component was included, and no structured qualitative interviews or dedicated qualitative data collection procedures were conducted.

Clinical outcomes were assessed quantitatively at baseline and after the 8-week intervention period. Given the pilot nature and limited sample size of the study, analyses of clinical outcomes were intended to provide preliminary estimates of potential changes and between-group differences rather than confirmatory evidence of intervention effectiveness. The findings are intended to inform the methodological design and outcome selection of a future adequately powered randomized controlled trial.

## 2. Methods: Participants, Interventions, and Outcomes

### 2.1. Study Setting

All pre-intervention (pre-test) and post-intervention (post-test) assessments were conducted at the Jerzy Kukuczka Academy of Physical Education in Katowice, Poland. Procedures involving the assessment of spinal mobility and the mechanical properties of soft tissues were carried out in the Laboratory of Body Structure and Posture Assessment. The laboratory is headed by Diana Celebańska, PhD, and operates within a quality management system compliant with the PN-EN ISO 9001:2015 standard (Warsaw, Poland, 2016.) [16] implemented at the Jerzy Kukuczka Academy of Physical Education in Katowice. Assessments and interventions were conducted under standardized conditions in accordance with approved research protocols and established measurement and therapeutic procedures.

Respiratory function assessments were performed in the Clinical Physiology Laboratory under the supervision of Marcin Sikora, PhD. This laboratory also operates within the quality management system compliant with the PN-EN ISO 9001:2015 standard [16] implemented at the Jerzy Kukuczka Academy of Physical Education in Katowice. All examinations and interventions were carried out under standardized conditions and in accordance with applicable measurement and therapeutic procedures.

### 2.2. Protocol Version, Registration, and Study Status

No formal version identifier was assigned to the operational protocol contemporaneously; this revised reporting version is dated 10 September 2026. Ethics approval was granted on 5 December 2024 (Resolution No. 2-XII/2024), before recruitment or study procedures. Recruitment ran from May to December 2025. The study was registered retrospectively with ANZCTR (ACTRN12626000148370) on 5 February 2026. No amendments affecting eligibility, allocation, interventions, outcomes, or analysis could be verified in the contemporaneous materials available for this report.

### 2.3. Eligibility Criteria

- The study included mothers aged 25 to 44 years who were raising one child with a congenital disability and who had held an official disability certificate for at least two years.
- Recruitment of mothers of children with disabilities was conducted through healthcare facilities, schools for children with disabilities, and information was disseminated via Facebook social media platforms.
- Eligibility also requires written informed consent. The original study records used the phrase ‘generally good health’ and a participant declaration of no exclusion criteria but did not provide a sufficiently operationalized list in the materials available for this revision; these vague criteria are not presented as reproducible eligibility rules.
- Participants were required to have a raw WHO-5 score below the maximum possible score (<25 on the 0–25 raw scale) and either mild musculoskeletal pain (VAS 1–4 on a 0–10 scale) or tension-related complaints and/or reduced mobility identified during functional assessment. The WHO-5 criterion indicated less than maximal self-reported well-being and was not a diagnostic threshold for stress or mental disorder.

### 2.4. Group Allocation and Choice of Comparators

A total of 45 mothers were assessed as eligible; 13 declined before allocation and 32 consented and entered the study. Participants were allocated to CST (*n* = 12), RT (*n* = 10), or CG (*n* = 10). The active groups represented two distinct non-pharmacological approaches; they were not combined. The no-intervention control provided a pragmatic comparator, although it did not control for attention or expectation.

Allocation was non-randomized. The principal investigator assigned participants according to enrolment sequence and the project delivery schedule: earlier enrollees entered CST so that eight weekly therapist-delivered sessions could be completed within the grant period; later enrollees entered RT or CG according to remaining capacity. Allocation was not based on baseline outcomes, but this procedure could still create selection bias and confounding. Participants, the CST therapist, most outcome assessors, and the analyst were not blinded; respiratory tests were performed by a separate investigator who was also not documented as blinded.

**Figure 1:**
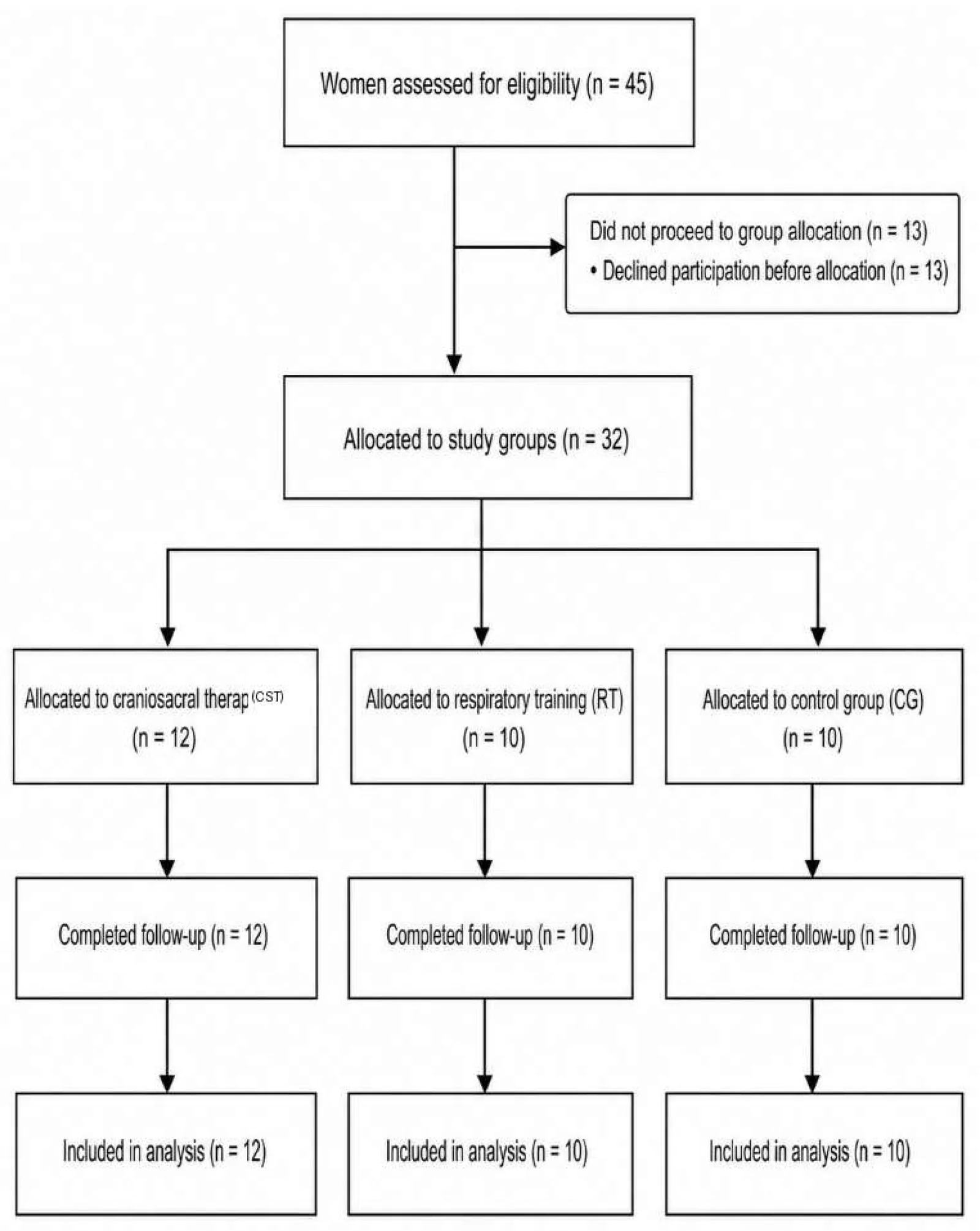
Flow of participants through the study. CST, craniosacral therapy group; RT, respiratory training group; CG, control group.

The intervention period lasted 8 weeks, after which the post-intervention assessment was performed. All assessments were conducted in the morning at the laboratories of the Jerzy Kukuczka Academy of Physical Education in Katowice, Poland. For each participant, all measurements were completed within a single day and were performed in an order intended to minimize the potential influence of fatigue. Participant flow through the study is presented in Figure 1.

### 2.5. Sample Size

No formal priori sample size calculation was performed because the study was designed as a pilot and feasibility project conducted within the framework of a time-limited institutional mini-grant. The sample size was therefore determined pragmatically based on recruitment feasibility, available project resources, and the capacity to deliver the interventions within the project timeframe. In particular, the CST intervention required eight individual 60 min sessions per participant, delivered once weekly over eight consecutive weeks by the same therapist, which limited the number of participants who could feasibly complete the intervention during the funding period. The final sample comprised 32 participants: 12 in the CST group, 10 in the respiratory training group, and 10 in the control group. Accordingly, the study was not powered to provide confirmatory evidence of intervention effectiveness, and analyses of clinical outcomes should be interpreted as exploratory.

### 2.6. Research Procedure

According to the study protocol, all measurements were performed before the intervention period (pre-test) and after its completion (post-test).

### 2.7. Feasibility and Acceptability Outcomes

Feasibility was summarized for all enrolled participants. Recruitment was 32 enrolled/45 eligible (71.1%); consent was 32/45 (71.1%); retention was 32/32 (100%); and outcome completeness was 32/32 (100%) for baseline and 8-week assessments. CST attendance was defined as sessions completed out of eight scheduled sessions, and RT adherence as diary-recorded sessions out of 112 prescribed sessions. Verified aggregate attendance and adherence values were not available in the records reviewed for this report and are therefore identified as unavailable rather than reconstructed. Fidelity was assessed indirectly from use of a common CST sequence and RT diary documentation; no formal fidelity checklist was used. No validated acceptability scale, prospective progression threshold, formal stopping rule, or systematic adverse-event instrument was prespecified. Retention and adherence are therefore treated only as indirect indicators of acceptability outcomes is presented in Table 1.

### 2.8. Outcome Hierarchy

Consistent with the pilot and feasibility focus of the present study, feasibility and acceptability outcomes were evaluated in terms of recruitment, consent, retention, intervention attendance and adherence, completeness of outcome assessments, intervention fidelity, withdrawals, and adverse events.

For the clinical outcomes, the hierarchy reported in the trial registry (ANZCTR; ACTRN12626000148370) was retained. The registered primary clinical outcomes were spinal mobility assessed using the IDIAG M360 system and respiratory function assessed using maximal inspiratory pressure (MIP), evaluated at baseline and after the 8-week intervention. Registered secondary outcomes included salivary cortisol concentration, myotonometric measures of tissue tone and stiffness, musculoskeletal symptoms, health-related quality of life assessed using the 36-Item Short Form Health Survey (SF-36), and psychological well-being assessed using the World Health Organization Five Well-Being Index (WHO-5).

Given the pilot nature and limited sample size of the study, analyses of these clinical outcomes were intended to provide preliminary estimates of potential changes rather than confirmatory evidence of intervention effectiveness. The schedule of enrolment, interventions, and assessments is presented in Table 2, the outcome hierarchy and analysis framework are summarized in Table 3, and the intervention and control conditions are summarized in Table 4.

## 3. Objective Outcome Measures (Pre–Post)

Objective outcome measures were assessed at baseline and after the 8-week intervention period. The objective assessment included spinal mobility, mechanical properties of the paraspinal soft tissues, respiratory function, sleep-related parameters, and salivary cortisol concentration.

Spinal mobility was assessed using the IDIAG M360 (SpinaMouse) system (IDIAG, Fehraltorf, Switzerland). The mechanical properties of the paraspinal soft tissues were assessed using the MyotonPRO device (Myoton AS, Tallinn, Estonia). Respiratory assessment included spirometry using the GANSHORN SpiroScout system (GANSHORN Medizin Electronic GmbH, Niederlauer, Germany), maximal inspiratory and expiratory pressure measurements using the RP Check respiratory muscle strength meter (Micro Medical Ltd., Rochester, UK), respiratory mechanics assessed using the forced oscillation technique with the Resmon PRO FULL (V3) system (RESTECH Srl, Milan, Italy), and body plethysmography using the MiniBox device (PulmOne Advanced Medical Devices Ltd., Ra’anana, Israel). Sleep-related parameters were assessed using the Nox T3 polygraphic system (Nox Medical, Reykjavík, Iceland). Salivary cortisol concentration was determined using a Salivary Cortisol ELISA assay at the Biochemistry Laboratory of the Institute of Sport Sciences, Jerzy Kukuczka Academy of Physical Education in Katowice, Poland.

### 3.1. Assessment of Spinal Mobility

Spinal mobility was assessed using the IDIAG M360 system (IDIAG, Switzerland), also referred to as the SpinaMouse, a non-invasive device designed to assess spinal curvature and mobility in the sagittal plane. All measurements were performed in the morning between 08:00 and 12:00 by the same investigator in the Body Posture Laboratory of the Jerzy Kukuczka Academy of Physical Education in Katowice, Poland.

Measurements were obtained along the spinal column from C7 to S3. Participants were assessed barefoot and without outer clothing to allow the device sensor to move directly along the spinous processes. In accordance with the standardized measurement protocol, three test positions were assessed: habitual standing, maximal trunk flexion, and maximal trunk extension.

During habitual standing, participants stood in their natural upright position with the gaze directed forward and the upper limbs relaxed alongside the body. For maximal trunk flexion, the feet were positioned approximately hip-width apart, the knees remained extended, the upper limbs were extended with the hands directed toward the floor, and the gaze followed the movement of the head and trunk. For maximal trunk extension, participants maintained the feet approximately hip-width apart and the knees extended, crossed the arms over the chest, and extended the trunk while maintaining the head in a natural position without excessive cervical extension.

Measurements were automatically recorded in degrees by the IDIAG M360pro 7.7.0 software. One measurement per position was obtained by the same investigator. Calibration procedures, assessor reliability, and repeatability coefficients were not documented in the materials available and are acknowledged as limitations.

### 3.2. Myotonometric Assessment

The mechanical properties of the paraspinal soft tissues were assessed using a MyotonPRO device (Myoton AS, Tallinn, Estonia). All measurements were performed by the same physiotherapist in the morning between 08:00 and 12:00 in the Body Posture Laboratory of the Jerzy Kukuczka Academy of Physical Education in Katowice, Poland. Participants were assessed in a relaxed prone position.

Measurement sites were selected according to the Fascial Manipulation concept described by Stecco and corresponded to the centers of coordination of the myofascial units involved in posterior trunk movement. The assessed regions represented the retro-collum (re-cl), retro-thorax (re-th), retro-lumbi (re-lu), and retro-pelvis (re-pv) myofascial units. Device settings, repetitions per site, calibration, averaging rules, and assessor reliability were not sufficiently documented to reconstruct and should not be inferred retrospectively.

Measurements were obtained bilaterally at sites corresponding to the C6, TH4, TH5, TH6, L1, and L5 spinal levels. At each level, the right side was assessed first, followed by the left side. A single measurement was obtained at each measurement site on each side by the same investigator.

The parameters analyzed in the present study were oscillation frequency (F, Hz), interpreted as an index of resting tissue tone, and dynamic stiffness (S, N/m), reflecting tissue resistance to deformation induced by a brief mechanical impulse.

### 3.3. Assessment of Respiratory Function

Respiratory function testing was performed by the same experienced investigator (M.S.), a physiotherapist qualified to perform pulmonary function testing. Measurements were conducted in the morning between 08:00 and 12:00, with participants in a fasting state and at least 2 h after waking. Participants were instructed to avoid alcohol consumption and strenuous physical activity for 24 h before testing. Respiratory assessments were performed with participants in a seated position and using a nose clip where required by the respective measurement procedure. Oscillometry was performed before spirometry.

#### 3.3.1. Oscillometry

Respiratory system impedance was assessed using the Resmon PRO V3 system (RESTECH Srl, Milan, Italy) in accordance with current European Respiratory Society (ERS) technical standards [17]. Measurements were performed with participants seated and breathing quietly through a mouthpiece while wearing a nose clip. The cheeks and floor of the mouth were supported to minimize upper-airway shunting. Measurements were repeated only when the recording was technically invalid or unsuccessful. The parameters analyzed were respiratory system resistance at 5 Hz (R5), reactance at 5 Hz (X5), and resonant frequency (Fres).

#### 3.3.2. Spirometry

Spirometry was performed using an ultrasonic GANSHORN SpiroScout spirometer (GANSHORN Medizin Electronic GmbH, Niederlauer, Germany) in accordance with the American Thoracic Society/European Respiratory Society (ATS/ERS) acceptability and repeatability criteria [18]. Participants were assessed in a seated position using a nose clip. The best acceptable maneuver was used for analysis. The analyzed parameters were forced vital capacity (FVC), forced expiratory volume in 1 s (FEV1), the FEV1/FVC ratio, and forced expiratory flow at 50% of forced vital capacity (FEF50).

#### 3.3.3. Respiratory Muscle Strength

Respiratory muscle strength was assessed by measuring maximal inspiratory pressure (MIP) and maximal expiratory pressure (MEP) at the mouth using an RP Check respiratory pressure meter (MD Diagnostics Ltd., Maidstone UK). Measurements were performed with participants seated and results were expressed in cmH_2_O. The starting lung volumes, number of attempts, acceptance and repeatability criteria, and rule used to select the analyzed value were not documented in the contemporaneous materials available for this report. The measurements were performed, but these procedural details cannot be reconstructed reliably; this limits reproducibility, particularly for MIP as a registered primary clinical outcome.

#### 3.3.4. Lung Volumes and Gas Transfer

Lung volumes were assessed using the MiniBox system (PulmOne Advanced Medical Devices Ltd., Ra’anana, Israel). The assessed parameters included residual volume (RV) and total lung capacity (TLC). Gas transfer was additionally assessed using the single-breath diffusing capacity for carbon monoxide (DLCO/TLCO). Measurements were performed with participants in a seated position in accordance with the standardized pulmonary function testing procedure.

### 3.4. Sleep Polygraphy

Sleep-related respiratory parameters were assessed using the Nox T3 portable polygraphic system (Nox Medical, Reykjavík, Iceland). The examination was performed overnight in the participant’s home. Before the recording, each participant received detailed instructions regarding correct placement and use of the device and procedures intended to minimize interference with the recording. The system recorded airflow, respiratory effort, oxygen saturation, body position, and snoring. After completion of the overnight recording, the device was returned to the laboratory and the recorded data were analyzed by the same experienced investigator (M.S.). Participants subsequently received an individual report of their examination. The available records do not document minimum valid recording duration, formal scoring rules, technical-failure handling, or scorer blinding; these omissions limit reproducibility.

### 3.5. Salivary Cortisol Assessment

Salivary cortisol concentration was assessed using morning saliva samples collected under fasting conditions. Participants provided saliva directly into three separate collection tubes. The samples were subsequently frozen and stored until biochemical analysis. Salivary cortisol concentration was determined using the SLV-2930 Salivary Cortisol ELISA assay (SLV-2930; DRG Instruments GmbH, Marburg, Germany). Laboratory analyses were performed at the Biochemistry Laboratory of the Institute of Sport Sciences, Jerzy Kukuczka Academy of Physical Education in Katowice, Poland. The same sampling procedure was applied at baseline and after the 8-week intervention period. Time relative to awakening, behavioural restrictions beyond fasting, medication and hormonal factors, storage temperature/duration, duplicate-assay reliability, detection limits, and a prespecified cortisol endpoint were not documented. A single morning sample at each time point cannot characterize chronic stress or the cortisol awakening response and is treated as exploratory.

## 4. Subjective Outcome Measures (Pre–Post)

Musculoskeletal symptoms were assessed using the Nordic Musculoskeletal Questionnaire (NMQ) across nine body regions: neck, shoulders, upper back, elbows, wrists/hands, lower back, hips/thighs, knees, and ankles/feet. NMQ-7 assessed symptoms during the previous 7 days and was treated as an exploratory pre–post symptom indicator. Because the six-month recall period extended substantially before the eight-week intervention, NMQ-6 was used only as descriptive contextual information concerning the broader history and anatomical distribution of symptoms and was excluded from analyses intended to represent change during the intervention. Binary symptom indicators were summarized using counts and proportions. The NMQ measures occurrence and distribution rather than symptom intensity.

Pain intensity was assessed separately using the Visual Analogue Scale (VAS). The scale ranged from 0 to 10, where 0 represented no pain and 10 represented the worst imaginable pain. Participants indicated the intensity of their perceived pain on the scale.

Psychological well-being was assessed using the World Health Organization Five Well-Being Index (WHO-5), a five-item self-report measure of subjective psychological well-being. Each item was scored from 0 to 5, resulting in a raw total score ranging from 0 to 25, with higher scores indicating better well-being. In the present study, the raw 0–25 score was used.

Health-related quality of life was assessed using the 36-Item Short Form Health Survey (SF-36). The questionnaire evaluates eight domains: physical functioning, role limitations due to physical health, bodily pain, general health perceptions, vitality, social functioning, role limitations due to emotional problems, and mental health. Higher domain scores indicate better health-related quality of life.

### 4.1. Description of Interventions

The study included two distinct non-pharmacological interventions: craniosacral therapy (CST) and respiratory training (RT). The interventions were delivered independently and were not combined within a single treatment arm. The control group (CG) did not receive either study intervention during the 8-week study period. The planned exploration comparisons included CST versus CG, RT versus CG, and CST versus RT. These comparisons were intended to generate preliminary estimates of potential between-group differences rather than to provide confirmatory evidence of comparative effectiveness.

#### 4.1.1. Craniosacral Therapy (CST)

Craniosacral therapy (CST) was delivered individually in a one-to-one therapist–participant format once weekly for 8 consecutive weeks, resulting in a total of eight sessions per participant. Each session lasted approximately 60 min. All sessions were delivered by the same physiotherapist (A.G.), a certified CST therapist, according to the same standardized intervention protocol throughout the study.

CST was based on gentle, non-invasive manual contact, described within the therapeutic approach as a “butterfly touch”, corresponding to approximately 5 g of applied force. The intervention involved gentle perceptual tactile contact intended to promote relaxation and a state of balanced tissue tension. No manual mobilization or manipulation techniques were performed. The intervention was standardized rather than individually tailored. Missed-session rescheduling, co-intervention restrictions, a formal fidelity checklist, and procedures to reduce therapist allegiance were not documented and are not added retrospectively.

Sessions were conducted between 12:00 and 21:00 in the same treatment setting using a therapy table. Participants remained comfortably dressed and rested in a relaxed supine position with the head supported by a pillow; a blanket was provided when required. Before the intervention, participants were informed that sensations of warmth or cold of varying intensity might occur during the session, although the intervention could also proceed without any particular sensory sensations.

Each session followed the same predefined sequence of therapist hand placements. The intervention began at the feet and subsequently involved the sacrum, lumbar region, diaphragm, superior thoracic aperture, cranial base around the foramen magnum, occipital region, temporal bones, frontal bone, and sphenoid bone, followed by completion of the session. The same sequence was applied to all participants during each of the eight sessions.

Following each session, participants were provided with mineral water and advised to maintain adequate hydration. They were also informed that transient emotional responses, such as irritability, could occur following the intervention. Participants were instructed to contact the therapist if such symptoms persisted for more than 72 h or at any time if they experienced concerns related to the intervention.

#### 4.1.2. Respiratory Training Program (RT)

Participants in the respiratory training group completed an 8-week home-based respiratory training program comprising resistive inspiratory muscle training (IMT), controlled breathing exercises, and breathing exercises combined with spinal movement. Inspiratory muscle training was performed using a POWERbreathe Plus device (POWERbreathe International Ltd., Southam, UK). Participants completed two sessions of 30 inspiratory efforts per day. Training resistance was individualized and progressively increased by one-quarter turn of the resistance adjustment knob once the participant was able to complete 30 consecutive inspirations without interruption. The precise baseline resistance/load, reassessment schedule, total duration of the slow-breathing component, and formal stopping criteria were not documented; the device and breathing elements should therefore be interpreted as a multicomponent program whose component-specific effects cannot be separated.

Before beginning the program, participants received instruction on the correct use of the device. The first three training sessions were supervised remotely, after which training was performed independently at home. Participants maintained training diaries documenting completion of the prescribed sessions, any symptoms experienced during training, and increases in training resistance.

Controlled breathing exercises consisted of a slow nasal inspiration, a brief breath hold of approximately 1–2 s, and a prolonged expiration, with a target inspiratory-to-expiratory time ratio of approximately 1:2. The exercises were performed in either a seated or quadruped position, with emphasis on lower-rib expansion, typically in sets of 5–10 repetitions. In the quadruped position, breathing was additionally combined with alternating spinal flexion and extension. No adverse events related to the respiratory training program were reported during the 8-week intervention period.

The amount and mode of therapist contact differed between the intervention groups. Participants in the CST group received eight individual, approximately 60 min face-to-face sessions with the same therapist, whereas the RT program was primarily home-based, with the first three training sessions supervised remotely and subsequent sessions performed independently. The control group received neither study intervention. Because of the nature of the interventions, participants could not be blinded to group allocation, and no attention-matched or sham control condition was used.

#### 4.1.3. Control Group (CG)

- Participants in the control group did not receive any intervention during the study period,
- In accordance with ethical principles, participants were informed about the opportunity to receive one of the therapeutic interventions after completion of the project.

### 4.2. Statistical Analysis

Statistical analyses were performed in R 4.3.2 after study completion. The primary quantities of interest were the feasibility proportions for recruitment, consent, retention, attendance/adherence, and outcome completeness. Clinical analyses were secondary and exploratory. The target exploratory contrasts were CST versus CG, RT versus CG, and CST versus RT for change from baseline to 8 weeks. The analyses estimated differences in observed change among participants as allocated; no causal estimand was prespecified, and the non-randomized design leaves residual confounding. The principal clinical analyses used change scores because these were the analyses conducted before revision. Baseline-adjusted or mixed models were not represented retrospectively as prespecified analyses.

For each outcome, change scores were calculated as the difference between the post-intervention and baseline values (Δ = post-intervention value − baseline value). The distribution of change scores was assessed using the Shapiro–Wilk test.

Within-group pre–post comparisons were performed using paired Student’s t-tests for variables meeting the normality assumption and Wilcoxon signed-rank tests for variables that did not meet this assumption. These tests described temporal change within groups and were not interpreted as evidence of an intervention-specific effect. Between-group differences in change scores were assessed using the Kruskal–Wallis test. When the overall test indicated a difference (*p* < 0.05), pairwise Mann–Whitney U comparisons were performed for CST versus RT, CST versus CG, and RT versus CG. Binary NMQ symptom indicators were summarized as counts and proportions rather than analyzed as continuous change scores.

For salivary cortisol, for which the assumptions for parametric analysis were met, within-group changes were assessed using paired Student’s *t*-tests, while between-group differences in change scores were assessed using one-way analysis of variance (ANOVA), followed by Tukey’s post-hoc test where appropriate.

To account for multiple comparisons within related outcome families, Holm adjustment was applied separately to the family of sleep-related outcomes and to the family of spirometric and oscillometric outcomes. No multiplicity adjustment was applied across the complete set of exploratory clinical outcomes; consequently, unadjusted *p*-values outside these families are descriptive and not confirmatory.

Effect sizes were calculated to complement statistical significance testing. Standardized mean change was calculated as d = mean(Δ)/SD(Δ), and rank-biserial correlation (r) was calculated where appropriate. Effect estimates and 95% confidence intervals are intended for the reporting of clinical results, where supported by the corresponding estimators. No clinical outcome results are presented in this protocol report. Given the small sample, all clinical estimates and *p*-values were interpreted cautiously and within an exploratory framework. No confounder-adjusted model or formal sensitivity analysis was prespecified; additional complex models were not introduced retrospectively. Age and baseline outcome value are identified as priorities for a future adequately powered trial.

All 32 enrolled participants contributed to the feasibility analysis and completed 8-week outcome assessment; no pre-post values were missing. The analysed clinical sample therefore equalled the full enrolled sample. No imputation, per-protocol exclusion, outlier exclusion, treatment-discontinuation analysis, or missing-data sensitivity analysis was required or performed.

## 5. Patient and Public Involvement

Patients and members of the public were not formally involved in the design, conduct, recruitment, or dissemination plans of this study. However, the study concept was informed by clinical observations and interactions with mothers caring for children with disabilities, who frequently reported high levels of stress, reduced well-being, musculoskeletal discomfort, and difficulties accessing supportive interventions. These experiences contributed to the development of research questions and the selection of outcomes related to quality of life, pain, muscle properties, and respiratory function.

Participants were not involved in determining the study design, recruitment procedures, or outcome measures. The burden associated with participation was considered by the research team during protocol development, and the intervention schedule was designed to maximise feasibility and acceptability for participants.

Patients and members of the public will not be involved in data analysis. The results of the study will be disseminated through scientific publications and conference presentations. Participants will be able to obtain a summary of the study findings upon request.

## 6. Ethics, Safety, Data Protection and Dissemination

The study protocol entitled “Assessment of the Effects of Craniosacral Therapy and Breathing Exercises (as Methods of Reducing Stress and Its Consequences) on Functional Status and Quality of Life in Women Raising Children with Disabilities” received approval from the Bioethics Committee of the Jerzy Kukuczka Academy of Physical Education in Katowice on 5 December 2024 (Resolution No. 2-XII/2024), before recruitment and the initiation of study procedures. All participants provided written informed consent before participation in the study. The study was subsequently registered with the Australian New Zealand Clinical Trials Registry (ANZCTR; ACTRN12626000148370) on 5 February 2026 and is classified as retrospectively registered.

Participants could report symptoms spontaneously to study personnel and were instructed to contact the CST therapist if intervention-related concerns occurred. Symptoms, withdrawals, and adverse events documented in the available study records were reviewed by the responsible investigator; no intervention-related adverse events were reported. A systematic adverse-event questionnaire, prospective severity or causality definitions, formal stopping rules, independent medical oversight, and standardized referral algorithms were not documented. Accordingly, the absence of reported intervention-related adverse events should not be interpreted as establishing intervention safety. Participants could withdraw at any time without affecting their care.

Data used for analysis were coded with participant identifiers, stored electronically, and accessible only to the investigator responsible for data management. No directly identifying information is reported. Processing and any sharing must follow the consent, ethics approval, institutional retention rules, and applicable GDPR requirements.

Study findings will be disseminated through scientific publications and conference presentations. Participants may obtain a summary of the study findings upon request.

## 7. Discussion

The presented project addresses the need to investigate feasible non-pharmacological approaches for supporting the psychophysical health of caregivers of children with disabilities. The study used a common intervention sequence and a consistent multidimensional assessment framework. However, incomplete documentation of several operational intervention and measurement parameters limits full reproducibility. Available evidence indicates that parents of children with intellectual and developmental disabilities are more likely to experience high levels of stress, anxiety, and depressive symptoms [19,20,21]. Chronic stress may negatively affect psychological well-being, social functioning, and quality of life among caregivers [22,23,24,25] and may influence functioning within the wider family system [26,27].

Interest has increased in non-pharmacological approaches to stress management, including relaxation, respiratory training, mindfulness-based interventions, manual therapies, and adapted physical activity [28]. Evidence and mechanisms differ across these approaches, and the present pilot evaluates CST and RT as separate, exploratory interventions within one methodological framework.

Previous studies have focused primarily on psychoeducational and cognitive–behavioral interventions, whereas the potential role of manual therapies in reducing chronic stress has been explored much less frequently. Systematic reviews of osteopathic manual interventions have suggested potential benefits in reducing stress levels, depressive symptoms, and disturbances in autonomic regulation; however, they have also emphasized the considerable heterogeneity of existing studies and the need for further well-designed research [29,30].

Similar caution applies to craniosacral therapy. Published work has often focused on subjective outcomes, including pain, quality of life, and psychological well-being. Some publications describe protocols or planned investigations incorporating physiological parameters and stress biomarkers [31,32], whereas completed pilot work in related manual interventions has reported exploratory psychometric findings [33]. Protocol publications are cited here as examples of planned methodological approaches, not as evidence of observed biomarker reductions. The evidence remains inconclusive and does not establish an effect of CST on stress biomarkers.

Combining subjective and objective measures has been proposed in research protocols investigating stress- and sleep-related interventions [31,32,34]. These protocol publications describe planned assessment strategies and are not presented as evidence of completed clinical effects. The present project similarly included standardized questionnaires, sleep-related measures, salivary cortisol, and instrumental biomechanical and respiratory assessments to explore the feasibility of a multidimensional outcome framework.

An important aspect of the project is the practical applicability of the proposed interventions. Both craniosacral therapy and respiratory training are non-invasive methods that may be relatively easy to implement in the daily lives of caregivers. If future adequately powered randomized controlled trials demonstrate their effectiveness, these approaches could potentially complement existing strategies supporting the psychophysical health of mothers of children with disabilities and contribute to preventive and supportive programs.

## 8. Strengths and Limitations of the Study

A strength of the study is the evaluation of two distinct non-pharmacological approaches, craniosacral therapy and respiratory training, within the same three-arm pilot and feasibility framework. Another strength is the inclusion of both subjective and objective indicators of psychophysical functioning. A common intervention sequence and consistent assessment framework were used; however, incomplete documentation of several operational parameters prevents a claim of full reproducibility.

One limitation of the study is its pilot nature and relatively small sample size, which restricts the generalizability of the findings and does not allow definitive conclusions regarding intervention effectiveness. The non-randomized allocation procedure may also have introduced selection bias and baseline imbalances between the study groups, as well as residual confounding by participant characteristics that could not be fully controlled in this small pilot sample. In addition, individual environmental and psychosocial factors associated with the daily lives of families raising children with disabilities may have influenced the assessed outcomes. The partial reliance on self-report measures introduces the possibility of reporting bias. The lack of assessor blinding represents an additional limitation and may have introduced measurement or observer bias, particularly for outcomes requiring investigator involvement.

In addition, the intervention groups differed substantially in the amount and mode of therapist contact. Participants receiving CST attended eight individual face-to-face sessions, whereas RT was predominantly performed independently at home after initial remote supervision. The absence of an attention-matched or sham control condition means that non-specific effects related to therapist contact, participant expectations, and treatment context cannot be separated from intervention-specific effects.

Furthermore, acceptability was not assessed using a dedicated satisfaction questionnaire or structured qualitative interview, and formal quantitative progression criteria for proceeding to a definitive randomized controlled trial were not prospectively specified. These limitations should be considered when interpreting the feasibility findings and should be addressed in the design of future studies.

Despite these limitations, the study provides a structured framework for evaluating two distinct non-pharmacological interventions in mothers of children with disabilities. The collected data may provide preliminary estimates and help identify potentially responsive outcome measures for future research. The study does not provide confirmatory evidence of intervention effectiveness or safety; its principal value is to inform future methodological procedures, outcome selection, documentation standards, and sample-size planning.

## Figures and Tables

**Table 1:** Feasibility and acceptability outcomes in the completed pilot study.

Domain	Definition/Denominator	Time Point	Source	Summary	Progression Rule
Recruitment	Enrolled/eligible	End of recruitment	Screening log	32/45 (71.1%)	No prospective threshold
Consent	Consented/eligible	Before allocation	Consent log	32/45 (71.1%)	No prospective threshold
Retention	Completed 8-week follow-up/enrolled	8 weeks	Study log	32/32 (100%)	No prospective threshold
CST attendance	Sessions attended/8 scheduled	Weekly; 8 weeks	Attendance record	Not available	No prospective threshold
RT adherence	Diary-recorded sessions/112 prescribed	Daily; 8 weeks	Training diary	Not available	No prospective threshold
Outcome completeness	Participants with baseline and follow-up data/enrolled	Baseline and 8 weeks	Dataset audit	32/32 for pre-post outcomes	No prospective threshold
Fidelity indicators	Standardized CST delivery; RT diary dose/progression	Throughout	Provider record/diary	Indirect only	No formal checklist
Acceptability	Completion, adherence, withdrawals, reported symptoms	8 weeks	Logs/diaries	Indirect indicators only	No dedicated measure
Safety/withdrawals	Events and withdrawals by group	Throughout	Logs/diaries	None reported	No formal stopping rule

**Table 2:** Schedule of enrolment, interventions, and assessments (adapted to the completed study).

Procedure	Baseline	Weeks 1–8	Week 8
Eligibility and consent	X		
Allocation	X		
CST or RT/control period		X	
Feasibility process data		X	X
Clinical outcome assessment	X		X
Adverse-event monitoring		X	X

X indicates the time point at which the respective procedure was performed.

**Table 3:** Outcome hierarchy and analysis framework. Clinical outcomes are exploratory; no single candidate primary clinical outcome was newly selected after data access.

Hierarchy	Variables	Assessor/Source	Time	Analysis
Primary feasibility	Recruitment, consent, retention, attendance/adherence, completeness, fidelity indicators, withdrawals, adverse events	Logs/diaries	Recruitment/baseline; throughout; 8 weeks	Counts, proportions, descriptive CIs where appropriate
Registered primary clinical	Spinal mobility; MIP	IDIAG M360; RP Check	Baseline and 8 weeks	Exploratory pre–post and between-group estimates
Secondary/exploratory	Myotonometry, spirometry/oscillometry, lung volumes/gas transfer, sleep polygraphy, cortisol, WHO-5, SF-36, NMQ-7 (exploratory pre–post), NMQ-6 (descriptive context), VAS	Specified instruments	Baseline and 8 weeks	Exploratory; direction depends on measure

**Table 4:** Summary of intervention and control conditions.

Group	Mode	Dose	Delivery/Progression	Monitoring
CST	1:1, face-to-face	8 × ~60 min; weekly	Certified CST physiotherapist; standardized hand-placement sequence	Attendance record; same provider/protocol
RT	Home-based multicomponent IMT + slow breathing + spinal movement	2 × 30 inspirations/day plus exercises; 8 weeks	POWERbreathe Plus; first 3 sessions remotely supervised; diary; resistance +1/4 turn after 30 uninterrupted inspirations	Diary sessions, symptoms, resistance
CG	No intervention	8 weeks	Usual activities; intervention offered after project	No attention matching

## Data Availability

No new data were created or analyzed in this study. Data sharing is not applicable to this article.

## Abbreviations

ANZCTR	Australian New Zealand Clinical Trials Registry
BMI	Body Mass Index
CG	Control Group
CST	Craniosacral Therapy
DLCO/TLCO	Diffusing Capacity of the Lung for Carbon Monoxide
ERS	European Respiratory Society
F	Oscillation Frequency
FEF50	Forced Expiratory Flow at 50% of Forced Vital Capacity
FEV1	Forced Expiratory Volume in 1 s
FOT	Forced Oscillation Technique
Fres	Resonant Frequency
FVC	Forced Vital Capacity
IMT	Inspiratory Muscle Training
MEP	Maximal Expiratory Pressure
MIP	Maximal Inspiratory Pressure
NMQ	Nordic Musculoskeletal Questionnaire
NMQ-6	Nordic Musculoskeletal Questionnaire, previous 6 months
NMQ-7	Nordic Musculoskeletal Questionnaire, previous 7 days
R5	Resistance at 5 Hz
RT	Respiratory Training
S	Dynamic Stiffness
SD	Standard Deviation
SF-36	36-Item Short Form Health Survey
VAS	Visual Analogue Scale
WHO-5	World Health Organization Five Well-Being Index
X5	Reactance at 5 Hz
